# Quality of Life as an outcome in Alzheimer's disease and other dementias- obstacles and goals

**DOI:** 10.1186/1471-2377-9-47

**Published:** 2009-08-25

**Authors:** Matthias W Riepe, Thomas Mittendorf, Hans Förstl, Lutz Frölich, Martin Haupt, Reiner Leidl, Christoph Vauth, Matthias Graf von der Schulenburg

**Affiliations:** 1 Department of Psychiatry and Psychotherapy II, Mental Health & Old Age Psychiatry, Ulm University, Ulm, Germany; 2 Center for Health Economics, Leibniz University Hannover, Hannover, Germany; 3 Department of Psychiatry and Psychotherapy, Technische Universität München, Munich, Germany; 4 Department of Geriatric Psychiatry, Central Institute of Mental Health, Medical Faculty Mannheim, University of Heidelberg, Mannheim, Germany; 5 Neurological Research Institute Düsseldorf, Düsseldorf, Germany; 6 Institute for Health Economics and Health Care Management, Helmholtz Zentrum München, Munich, Germany

## Abstract

**Background:**

The number of individuals at risk for dementia will probably increase in ageing societies as will the array of preventive and therapeutic options, both however within limited economic resources. For economic and medical purposes valid instruments are required to assess disease processes and the efficacy of therapeutic interventions for different forms and stages of illness. In principal, the impact of illness and success of an intervention can be assessed with biomedical variables, e.g. severity of symptoms or frequency of complications of a disease. However, this does not allow clear judgement on clinical relevance or comparison across different diseases.

**Discussion:**

Outcome model variables such as quality of life (QoL) or health care resource utilization require the patient to appraise their own well-being or third parties to set preferences. In Alzheimer's disease and other dementias the evaluation process performed by the patient is subject to the disease process itself because over progress of the disease neuroanatomical structures are affected that mediate evaluation processes.

**Summary:**

Published research and methodological considerations thus lead to the conclusion that current QoL-instruments, which have been useful in other contexts, are ill-suited and insufficiently validated to play a major role in dementia research, decision making and resource allocation. New models integrating biomedical and outcome variables need to be developed in order to meet the upcoming medical and economic challenges.

## Background

Prevalence of Alzheimer's disease and other dementias increase in the aging societies of the Western hemisphere. This puts an ever increasing burden on the health care systems. Considering increasing treatment options over almost the whole spectrum of diseases effective spending of the resources of health care systems is warranted to provide a fair if not comprehensive care. Hence payers, physicians and patients will need to weigh costs and benefits of future interventions much more carefully. The "burden of illness" and "quality of life" convey greater meaning and more direct relevance across a wide spectrum of diseases and illnesses than abstract clinical or scientific parameters, and such dimensions will gain greater appeal for a larger community involved in future decisions.

Biomedical measures are appropriate to determine the consequences of disease on specific medically relevant symptoms. For oncological diseases this may include the size of the tumor or the number and region of metastases. In order to assess the impact on the patient's everyday life these biomedical measures need to be supplemented by other measures relating to everyday life such as the quality of life or pain. For dementing disorders specific symptoms of disease such as cerebral atrophy can be measured in much the same way as the size of a tumor. Beyond this, several symptoms of dementia, e.g. the impairment of memory, can be measured with standardized instruments, whose relevance for everyday life is obvious. Over the course of Alzheimer's dementia the burden of disease is not primarily reflected by diminishing cognitive functions of patients, but also by a variety of behavioural problems and physical handicaps. There is, however, a great deal of heterogeneity regarding the manifestation of, and the coping with these non-cognitive symptoms. Therefore more comprehensive instruments are required to take account of such causes and their effects within the context of family or professional care.

Quality of Life (QoL) measurements require the patient's self-assessment of his or her fulfilment and impairment in everyday life. Sometimes seemingly negligible causes lead to major handicaps, and severe changes regarding the quality of life and resource usage, whereas severe medical problems need not be associated with apparent subjective consequences. These observations suggest that data on subjective relevance and economic outcomes should be included in clinical studies in order to prepare the ground for future cost-effectiveness models which will need to include the subjective and social impact of illness and interventions. [1].

We will address several moot points regarding the use of "quality of life" and other concepts and instruments, which have been developed for other target groups, but are now employed in the context of dementia. We hope to prevent premature conclusions based on the uncritical administration of such scales in patients with dementia, and we argue for the development of adequate tools.

## Discussion

### Biomedical instruments for assessing dementia

Dementia is a frequent disorder in the elderly and its prevalence increases with age [2]. The most frequent cause is AD. At onset of AD the medial temporal lobe is affected [3] resulting in episodic memory deficit as the early clinical hallmark [4]. As the disease spreads, other brain regions are affected as well. The parietal cortex mediates functions such as spatial orientation and visuospatial functions [5,6], the frontal cortex executive functions, planning, attention, and working memory [7-9]. Spread of AD beyond the temporal lobe thus is characterized in functional terms by accruing deficits of spatial orientation, attention and executive functions as well as working memory and language [4] beyond initial temporal lobe type memory deficits. This can be visualized using advanced imaging methods [10,11].

For Clinical Study purposes test batteries are used, most commonly the Mini Mental Status Examination (MMSE) [12,13] and the Alzheimer's Disease Assessment Scale (ADAScog). Cognitive scales such as the MMSE or the cognitive sub-scale of the ADAScog mingle the results of several cognitive functions into one composite score. While any of the assessed symptoms might be present at one time point or the other during the course of an individual's disease they will not be present all at once at any given stage of disease. Moreover, the dynamic range for observation of change is not the whole band of the scale but a small margin centered around the observed score. Unfortunately, the sensitivity to change of these scores is not linear over the course of disease. As recently reviewed [14] it often is argued that a certain score difference, e.g. a 4-point difference in the ADAScog score, represents a meaningful clinical outcome. However, this judgement remains arbitrary and does not reflect clinical observations of large score differences with hardly any differences in everyday life and vice versa.

### Impact of disease in terms of Quality of Life (QoL)

The concept of QoL relates to the 1947 definition of 'health' by the World Health Organization (WHO) as being a state of complete physical, mental, and social well-being [15]. In a similar fashion, Lawton characterized five domains pertaining to QoL for subjects with dementing illnesses to comprise the same areas as in people in general (cognitive functioning, ability to perform activities of daily living, being able to engage in meaningful time use, social behavior, as well as a favorable balance between positive emotion and absence of negative emotion) [16]. As QoL refers to all aspects of a patient's life, it can provide complementary and valuable information on the patient's self perception of health and treatment impact. Therefore, QoL is suggested in many disease areas as an important outcome to evaluate new treatments.

Considering the subjective nature of the QoL concept it is generally agreed that any appraisal thereof at best should rely on the perception of the individual to be looked at. The use of proxies (e.g relatives or a nurse) to measure QoL has inherent obstacles, such as personality characteristics of the proxy, the nature of the relationship, the time spent with one another, and the level of impairment. Frequently, proxy appraisal of the patient's QoL are disparate to the patients own evaluation [17]. Moreover, discrepancies between dementia patients' and their caregivers' ratings of the patients' quality of life are associated with increased levels of caregiver burden, rather than lower levels of patients' functioning alone [18,19] and thus are not only related to the disease process in the patient but external and internal factors of the proxy. The proxie's experiences of depression and burden might also negatively affect proxies' assessments of QoL [17]. Despite these observations this procedure has been used when patients are too severely impaired to complete the rating themselves. At best, proxy-ratings can be considered as complementary information for self-ratings but not as a substitute. Despite these difficulties and need for further methodological research [20], QoL has become an important dimension of AD therapeutic research [21] and health economic analyses in this area.

There are different concepts to measure a health-related quality of life (QoL). Rather than qualifying the approaches purely as either medical or economic, a methodological classification would focus on the major components used in describing and valuating QoL of dementia patients (Table 1). For measuring QoL of those who provide care for dementia patients (e.g., relatives) Table 1 without the proxy category would most likely be adequate. All approaches to value QoL require a descriptive element. (The literature mentions valuations based on numerical ratings alone, however, without a descriptive basis their valuation lacks any relation to aspects of the health status).

**Table 1:** Classification of approaches to describe and valuate QoL according to the person surveyed

Person surveyed or person defining	Measures to describe QoL		Procedures to valuate QoL	
	
	Generic	Disease-specific	Externally defined ^a^	Preference-based ^b^
Patient	I	II	**-**	1

Proxy	III	IV	**-**	2

Scientist	**-**	**-**	3	**-**

Population	**-**	**-**	**-**	4

^a ^e.g. a sum score

^b ^especially according to the procedures of VAS, TTO and SG, enabling the calculation of QALYs

### Quality of Life – Utility Measure in Health Economics

In economic cost-utility studies generic descriptions of QoL are combined with preference-based valuations. From an economic perspective, the preferences of the individual are the key criteria to assess whether goods or services can be considered "efficient"' from the perspective of the consumer (for most consumers, even the most cost-effectively produced shoes would be worth little if it were only left ones). Economists would suggest eliciting the preferences of those individuals affected by an intervention. When looking from a medical point of view, this would definitely be the patient. As in dementia the choice of the approach may be restricted by the capabilities of the patient, one would have to consider surveying a proxy (e.g., a relative) instead of the patient in cases where he would be unable to articulate his preferences but the use of this approach may be limited (cf. above).

Yet there are also other positions in the discussion with respect to the preferences to be used for valuation. When the question is about which services are to be covered in a national or statutory health service, some economists argue that it is the general population which is affected and that, accordingly, their preferences should be used for valuation (which is then typically derived via given pre-defined standardized health states in interviews or surveys). This approach, however, is distorted given the lack of knowledge about Alzheimer's disease in the general population [22-25]. The key problem in valuation, in any case, is to give a summary statement on QoL that integrates all different aspects of health at stake. Any answer to this problem should be scientifically well motivated. A simple solution is to use a pre-defined algorithm instead of preferences, e.g. a sum score across a health profile. Yet, this might only reflect the judgement of the constructor of the sum score. Obviously, constructors, the general population, the proxy or the patient himself may not assess a health state, such as e.g. mild dementia, in the same way. In the end, the choice of the method to integrate all health aspects will also depend on the type of decision that waits to be supported by the QoL data but proceeding in such a way somewhat forecloses a later result. Furthermore, the methodological usefulness of the approach used to describe and valuate QoL must be critically considered. The most important criteria for psychometric testing include reliability, validity and responsiveness [26]. For any measurement of QoL that claims to rely on scientific grounds it is necessary to show that the instrument used is psychometrically appropriate for the target group of patients or population analyzed – the foundation of what is being measured, and how well this is being achieved, is lacking otherwise. Accordingly, respective methodological pilot studies are required as long as the respective properties of the measurement instrument cannot be quoted from the literature.

In clinical research, one of the important differences of different QoL measures is measuring health status or utility of different health states. Health status measures describe functioning and the impact of illness on health. They are generally subdivided into generic and disease-specific measures. The generic measures are intended for general use and suitable for wide range of patient groups. Measuring general health status is important because it shows the extent to which interventions really make a difference to a patient's overall life and helps to quantify the relative effects of different interventions for patients with different diseases. The SF-36 and the EQ-5D instruments are the most frequently used generic QoL measures worldwide, while only for the EQ 5D broadly acknowledged valuation procedures are available to elicitate corresponding utilities. Typically one of these generic measures in clinical research is combined with one or more disease-specific measures. The latter aim at emphasizing problems specific to patients with the disease in question (e.g. EORTC QLQ-C30 or FACT-G in cancer research or the QOL-AD focusing on Alzheimer's disease). Compared with generic measures, disease-specific measures normally are more sensitive and responsive to the changes in the assessment of QoL of specific patient groups.

Utilities measures describe utilities or quality-of-life weights to health states derived by either direct or indirect utility measures. Direct utility measures include visual analogue scales (VAS), standard gamble (SG), and time trade-off (TTO) techniques. VAS asks patients to indicate on an e.g. 10 cm Likert scale the position of their current state, and also to mark positions corresponding to various scenarios. SG attempts to estimate patient's preferences under uncertainty, where the uncertainty involves a risk of death or some other outcome. On the other hand, TTO measures patients' preferences under certainty. The patients are asked to indicate whether they would choose one year in perfect health or one year with impaired health. SG and TTO have been found to be practical on most populations with TTO being a commonly used substitute for the SG method and vice versa. Some QoL instruments were developed to produce a series of health utilities, such as the Quality of Well-Being scale (QWB), the Health Utilities Index (HUI) and the EQ-5D from the EuroQoL group. They are referred to as being generic or indirect utility measures and in practice the generic utility measures are widely used mainly due to their ease in use: The answers of only few questions result in a QoL-utility. For example using the EQ-5D patients have to answer five questions, one question for one dimension of QoL (mobility, self-care, usual activities, pain/discomfort, and anxiety/depression), and give an expression of their current state of QoL via the VAS.

These utility measures assign values or utilities for health states from 0.0 to 1.0, where 0 is defined as death and 1 is defined as the best possible health state. The health state utilities are used to calculate quality-adjusted life-years (QALYs) which are applied in cost-utility or cost-effectiveness analysis. Based on health-related quality of life and survival for the patient, QALYs can assess the extent of the benefits gained from interventions. During that approach not only gained life time but also quality of that time gained is combined and it is defined that there exists the possibility of trade off between these two aspects. When the QALY is used in different indications and/or interventions, cross-comparisons can be made to describe the relative benefit for patients. When combined with the costs of providing the interventions, decision makers can understand the relative cost-effectiveness of different interventions. When resources are scarce one can argue that those interventions should be reimbursed which provide the best incremental cost-effectiveness ratio.

Regardless of whether a method is used that asks for a direct appraisal by the patient or that assesses of preferences these approaches implicitly assume that the patient is in a position to perform a valid appraisal of his or her own situation and that a sufficient knowledge about the disease is at hand.

### Deficits of judgement and decision-making in patients with dementia

Competent choices require awareness of competences and restrictions and a deliberate judgement on well- or ill-being despite present and future deficits. Lesions or malfunctioning of the frontal and prefrontal cortex foster a lack of awareness of deficits and impairments and ultimately the inability of formulating a free will [27]. A recent functional imaging study demonstrated this even in a semi-quantitative fashion. Decreased awareness was positively correlated with perfusion deficits in frontal regions [28]. It is unclear yet, whether impairment of deficit awareness is predominantly associated with frontal cortex malfunction or overall severity of central nervous system disease. While some studies suggest that the former is true [29] other studies suggest the latter [30-33]. Impaired awareness of deficits has been associated with malfunctions of the right hemisphere [34], the temporo-parietal junction [35], the right prosubiculum of the hippocampus [34], the parietal cortex [36], and the anterior cingulate gyri [37]. Thus a widespread network is involved in the awareness of deficits. This network is affected early on during progressing AD or other dementias although the pattern is unpredictable and subject to the specific disease and other internal and external factors in the patient, e.g. comorbidities.

Patients with mild dementia [27,34,38-42] and even those with Mild Cognitive Impairment (MCI) [43] frequently demonstrate decreased awareness of their cognitive impairments and change of behaviour. In particular, decreased awareness of deficits manifests as poor awareness of deficits in activities of daily living (ADL) [30]. Compared to their proxies, AD and MCI patients underestimate their deficits [36,44]. Moreover, however, family informants may also fail to recognize memory problems in subjects subsequently found to have dementia [45]. Patients' awareness of deficits is associated positively or negatively with age, gender, pre-morbid education and socioeconomic status in a complex fashion [31,46]. In general, awareness of deficits seems to decrease with an increased severity of dementia [30-32]. Awareness of deficits is modulated by even sub-syndromal depression, anxiety, psychosis, and apathy [28,31,47]. This modulation is subject to pre-morbid capacities as depressive symptoms and awareness were found to be positively related in high, but not low, cognitive reserve groups [48].

Standard approaches to judge the future impact of deficits on subjective well-being are also impaired in AD. AD patients perform less well in gambling tasks than elderly controls and show impairments in cognitive estimation [49]. Psychopharmacological and pathological evidence supports the concept of a 'cholinergic component' of conscious awareness [50]. In AD, a cholinergic deficit has been established many years ago [51] so that for this disease in particular the ability of the patient to undertake a deliberate choice is questioned.

On these grounds it can be expected that patients with mild AD have difficulties in taking decisions in every-day life situations, both in cases of ambiguity (information on probability is missing or conflicting, and the expected utility of the different options is incalculable) and in cases of risk (outcomes can be predicted by well-defined or estimable probabilities) [52]. Moreover, AD patients frequently change between strategies so that decisions are given in a random fashion which precludes the development of a consistent response pattern over time [53].

### Where to go?

In a summary recommendation aiming at patients after reviewing studies for dementia drugs, The American College of Physicians & The American Academy of Family Physicians concluded that outcomes related to QoL were studied (and hence, captured) less frequently than other outcome measures, and did not show consistent improvements attributable to drugs analyzed. Considering that dementia studies do not use survival as endpoint but rather employ intermediate surrogate endpoints, this is a remarkable statement. This enhances the need to elaborate on difficulties in accurately measuring QoL for this patient group [54]. Methodological problems are pinpointed when it comes to measuring the impact of drug treatment on QoL of dementia patients in terms of QALYs (Table 2).

**Table 2:** Appraisal of the internal validity of the QALY-endpoint in six cost-utility studies in the HTA on dementia drugs [56]

Product assessed (study)	Internal validity	Comment on generation of QALYs
Donepezil [58]	Unacceptable	Based on the Index of Health Related Quality of Life (IHQL) which was not validated for valuing cognitive impairment, and no rationale was given for the IHQL values used
Donepezil [59]	Unclear or unknown	Not stated
Rivastigmine [60]	Unacceptable	Based on the IHQL (see above)
Galantamine [61,62]	Unclear or unknown	Derived from [63] (pre – full time care at 0.60, full time care at 0.34)
Memantine [64]	Unclear or unknown/unacceptable	Insufficient detail found in the study

A few key questions which type of studies might be able to answer some questions appropriately can be taken from a current British consensus paper on good health economic modelling standards [55]: "Are the utilities incorporated into the model weights (utilities) appropriate? Is the source for the utility weights referenced? Are the methods of derivation for the utility weights justified?"

In their health technology assessment report on dementia drugs for the U.K. National Institute for Health and Clinical Excellence (NICE) Loveman et al [56] have evaluated the use of the QALY-endpoint in six cost-utility studies. Not one of the studies investigated achieved the label of being internally valid at this point, some of them lacking transparency, some lacking appropriate measurement instruments, and others using too simple assumptions (table 2). Next to the published literature, the industry submissions on the drugs investigated in this study were evaluated according to the quoted Philips et al. (2006) standards [55] with respect to the utility measurement. Again, no classification as 'internally valid' could be achieved.

It can thus be concluded that large gaps exist between published measurements of QoL in terms of utility and the quality standards required by guidelines. This conclusion is further supported by a consensus statement on measuring treatment benefits in dementia, in which the International Psychogeriatric Association has stated that health utility measures are not validated satisfactorily in dementia and that this calls into question previous health economic analyses [57].

These sobering findings on QALY measurements in dementia patients would further worsen if the guidelines' requirements were extended for the above claimed need to show the usefulness of the measurement instruments used according to the usual psychometric testing criteria which were not met by the quoted studies. Future research, [56] concluded accordingly, should include information on the quality of the outcome measures used as well as the need to establish QoL instruments for dementia patients.

## Summary

**Figure 1:**
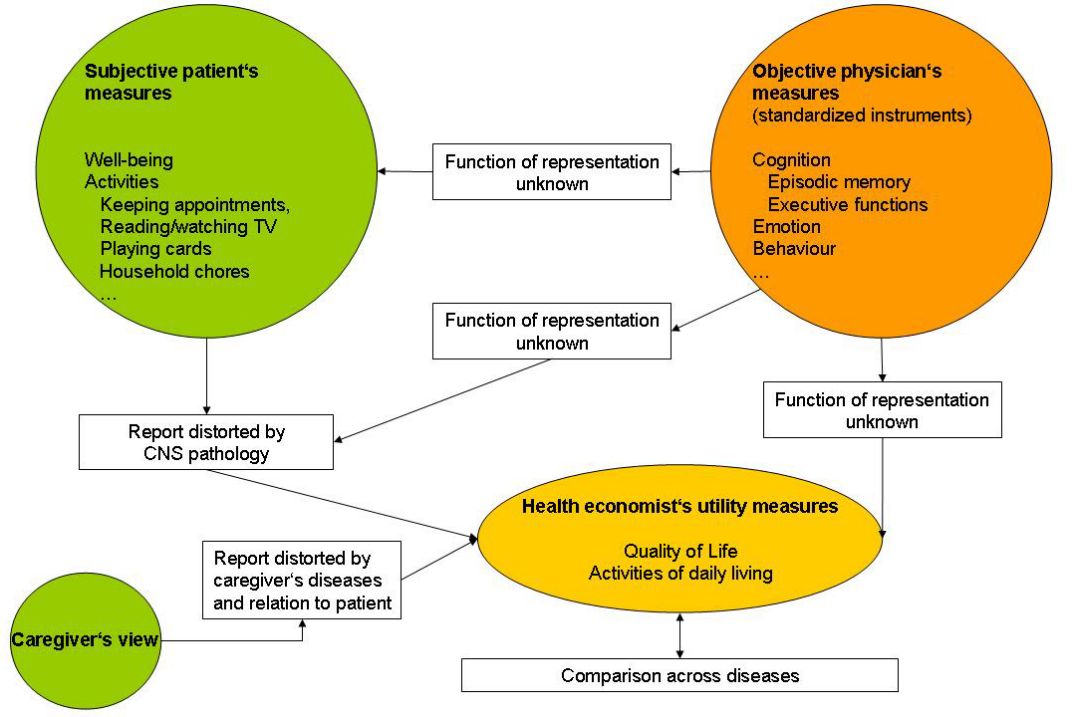
**Measures relevant for dementias**.

We conclude that current outcome model variables, especially quality of life measures, are well suited and established for non-dementing diseases but at present are not fully suited to be rested upon in medical or health economic analyses of disease impact or therapeutic interventions in the dementias. The function of representation of objective measures as obtained by biomedical assessments or proxy measures in their relation to everyday life or instruments of quality of life assessments is elusive, yet (Figure 1). In consideration of the increasing prevalence of Alzheimer's disease and other dementias in old age an integrative model of biomedical and outcome model variables is warranted to face the upcoming medical and health economic challenges.

## Competing interests

The authors declare that they have no competing interests.

## Authors' contributions

MWR was involved in drafting the manuscript, interpretation of the literature and critical revision. TM was involved in drafting the manuscript, interpretation of the literature and critical revision. HF was involved in drafting the manuscript, interpretation of the literature and critical revision. LF was involved in interpretation of the literature and critical revision. MH was involved in interpretation of the literature and critical revision. RL was involved in drafting the manuscript, interpretation of the literature and critical revision. CV was involved in interpretation of the literature and critical revision. MVDS was involved in interpretation of the literature and critical revision. All authors read and approved the final manuscript.

## Pre-publication history

The pre-publication history for this paper can be accessed here:


